# Jurassic fish choking on floating belemnites

**DOI:** 10.1038/s41598-025-00163-7

**Published:** 2025-05-08

**Authors:** Martin Ebert, Martina Kölbl-Ebert

**Affiliations:** Department of Earth and Environmental Sciences, Ludwig-Maximilians-Universität München, Luisenstr 37, 80333 Munich, Germany

**Keywords:** Solnhofen Archipelago, Jurassic fish, *Tharsis*, Belemnite, Predation, Choking on floating objects, Palaeontology, Behavioural ecology

## Abstract

*Tharsis*, an extinct genus of ray-finned fish (Actinopterygii) in the Late Jurassic, has but tiny teeth and is considered a micro-carnivore/visual zooplanktivore. A recent review of collection material, however, uncovered several specimens of *Tharsis* from the Late Jurassic (ca. 152 Ma) Plattenkalk deposits of the Solnhofen Archipelago with belemnites wedged in mouth and gill apparatus. In all cases, the rostrum reexits through the gill apparatus, whereas the broad phragmocone of the belemnite is firmly lodged in the mouth opening. Apparently, these micro-carnivore fish were in the habit of sucking remnants of decaying soft tissue or overgrowth such as algae or bacterial growth from floating objects, but when a streamlined floating belemnite rostrum accidentally was sucked into the mouth, they were no longer able to get rid of these deadly objects.

## Introduction

Modern-day fish are suffering from the increasing pollution of the seas with human products they consider to be food. Such dangerous alien objects range from plastic straws to toys and bottles^1–3^. Plastic in water is soon overgrown by algae and bacteria. Algae-coated plastic seems to fool fish by its odour into identifying plastic as food, and it may be ingested by hundreds of marine species^4^. A special problem these days is bottle-shaped plastic waste^5^, which some fish probably confuse with prey fish due to its shape. Here we report objects that were floating in the Late Jurassic Sea of the Solnhofen Archipelago, some 150 million years ago, which, due to their shape, were deadly to the fish of their time.

The fossils of the marine Plattenkalk deposits of the Solnhofen Archipelago are well known for their outstanding preservation of complete, articulated fossils^6^, especially vertebrates from the early bird *Archaeopteryx* to various fishes, often with soft part preservation such as skin, muscles, or gut content^7,8^. The fine lamination of the Plattenkalk deposits indicates the absence of benthic life in the basin facies of the Solnhofen Archipelago. Only in some of the Plattenkalk basins a few of the laminated beds contain rare benthic organisms and/or their traces, e.g. ophiuroids or gastropods. Possible causes for hostile conditions include oxygen depletion or hypersaline conditions^6^.

True teleosts (sensu Arratia 2015^9^), to which the specimens of *Tharsis* Blainville, 1818^10^ described here belong, exist since the early Jurassic. In the Late Jurassic, teleosts were micro-carnivores or ate small fish^11–13^ or crustaceans^14^, whereas large predatory fish, able to feed on larger fish, came from other groups of the Actinopterygii (Halecomorphi such as Caturoidea^15^ and *Callopterus*^16^, or Teleosteomorpha such as the Aspidorhynchidae^17^ and Pachycormiformes^18^). Among Late Jurassic fish, only large sharks are known as predators on belemnites^19^.

## Context and data

The genus *Tharsis* is among the most common fish in the Eichstätt and Solnhofen Basins of the Solnhofen Archipelago, with around 26% of the entire fish fauna^20^. In these two localities, juvenile and subadult specimens of *Tharsis* are found slightly more frequently than adult specimens, whose maximum size reaches around 27 cm total length^21^.

The gastrointestinal tract is well filled in many *Tharsis* specimens, but no recognizable fish or crustacean remains have been identified in this genus despite hundreds of examined specimens – in contrast to other Teleostei in the Solnhofen Archipelago where fish or crustacean remains are often recognizable^11,13,14,22,23^. This fact, together with the but tiny teeth in the genus *Tharsis*^24,25^, led to the general assumption that *Tharsis* only ate tiny foodstuff^13^. However, according to M. Wilson (pers. comm. 2015), because of the lack of fine, long gill-rakers and other filter-feeding adaptations, *Tharsis* apparently was no filter feeder, but rather a micro-carnivore/visual zooplanktivore selecting individual prey items in the water column and engulfing them using oral suction^11^.

Consequently, one would not expect an encounter of belemnites as prey for *Tharsis* – and yet, there are several such incidents documented in the fossil record, which – such is the nature of the fossil record – ended deadly for the presumed predator.

Belemnites are rare in the Plattenkalk basins of Eichstätt and Solnhofen^26^, where the described specimens were found. A rough survey of the most important collections revealed around 120 belemnites compared to some 15,000 fish. Like most of today’s Teuthida, belemnites (s.str.) of the Upper Jurassic of Southern Germany are reported to have preferred the open sea^26,27^ and they were possibly among the first to disappear when the oxygen content in shallow basins (such as most of the basins of the Solnhofen Archipelago) fell below a critical threshold^28^. According to Schweigert^27^, and Fuchs^26^, belemnites did not live in the waters of the Solnhofen Archipelago, and only dead individuals, still with the gas-filled phragmocone attached to the rostrum and thus floating, were washed in from the open Tethys Ocean further south. Indeed, almost all belemnites found in the Plattenkalk basins of Eichstätt and Solnhofen still have their phragmocone attached, and in many cases this phragmocone is also overgrown with bivalves (in 46% of the observed specimens), which means that these belemnites continued to float in the water column for quite some time after death and decay of the soft tissue. These bivalves attached to the belemnites are *Liostrea* or *?Pseudomytiloides* who in the Solnhofen Archipelago led a pseudo-planktonic way of life, attaching themselves to floating objects^29^. Settling on hard-ground at the sea floor, such as e.g. belemnite remains already sunk to the ground, was impossible, since conditions at the bottom of the Plattenkalk Basins of Eichstätt and Solnhofen were so hostile to life in general that bivalves were unable to survive^6^. Consequently, bivalves are rarely found in the Plattenkalk Basins of Eichstätt and Solnhofen, and if so only attached to objects such as plant remains, belemnites or ammonite shells able to drift in the water column. A few exceptions of this rule are presumed to have detached and fallen off from such floating objects^29,30^. The hostile conditions at the sea floor also make it highly unlikely that fish such as *Tharsis* were able to look for food on the seafloor^6^.

In rare cases, belemnites in the Solnhofen Archipelago are also found incorporated in larger rafts together with other fossils (sponges, ammonites, bivalves, further belemnites)^31^.

If epifauna on a belemnite is present, in nearly all cases (41 of 43 specimens) only the phragmocone of the belemnite is covered with bivalves. Only in two cases we found bivalves on the phragmocone as well as the rostrum. There may be several possible reasons for this preference. In a floating belemnite carcass, the heavy rostrum hangs downward and the gas-filled phragmocone is at the top, thus more exposed to light, which the bivalve larvae may simply prefer on a hardground raft. Another reason could be that the rostrum is calcitic and the phragmocone aragonitic, leading to differences in surface structure as well as chemical behaviour. Or soft tissue lasted longer on the rostrum, which in turn could also be a reason why the *Tharsis* specimens were interested in the floating belemnite remains.

Most belemnites of the Plattenkalk deposits in the Solnhofen Archipelago belong to *Hibolithes hastatus* (Montfort, 1808)^32^. Some rare, needle-shaped belemnites belong to *Raphibelus acicula* (Münster, 1830)^33^ (see Fuchs 2015^26^).

In all observed specimens of *Tharsis* with belemnite in the mouth, the belemnite belongs to the genus *Hibolithes* with a rostrum of about 6 cm plus a phragmocone of another 6 cm in length.

### Specimens

**Fig. 1:**
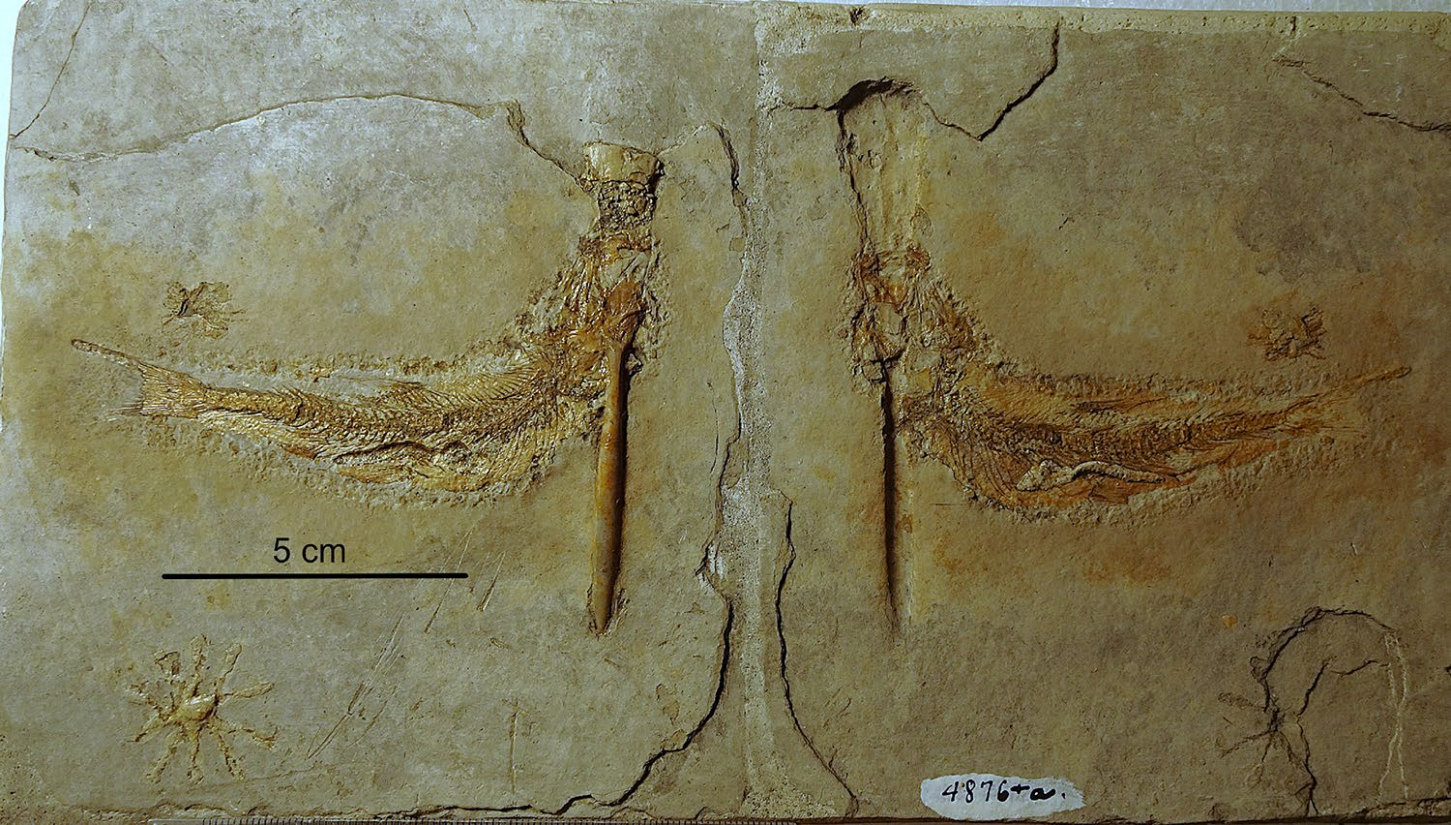
*Tharsis* with belemnite lodged through mouth and gill apparatus (CM4876) from Solnhofen, Bavaria, Germany. Part and counterpart glued together to form one continuous slab. (Photos M. Ebert).

**Fig. 2:**
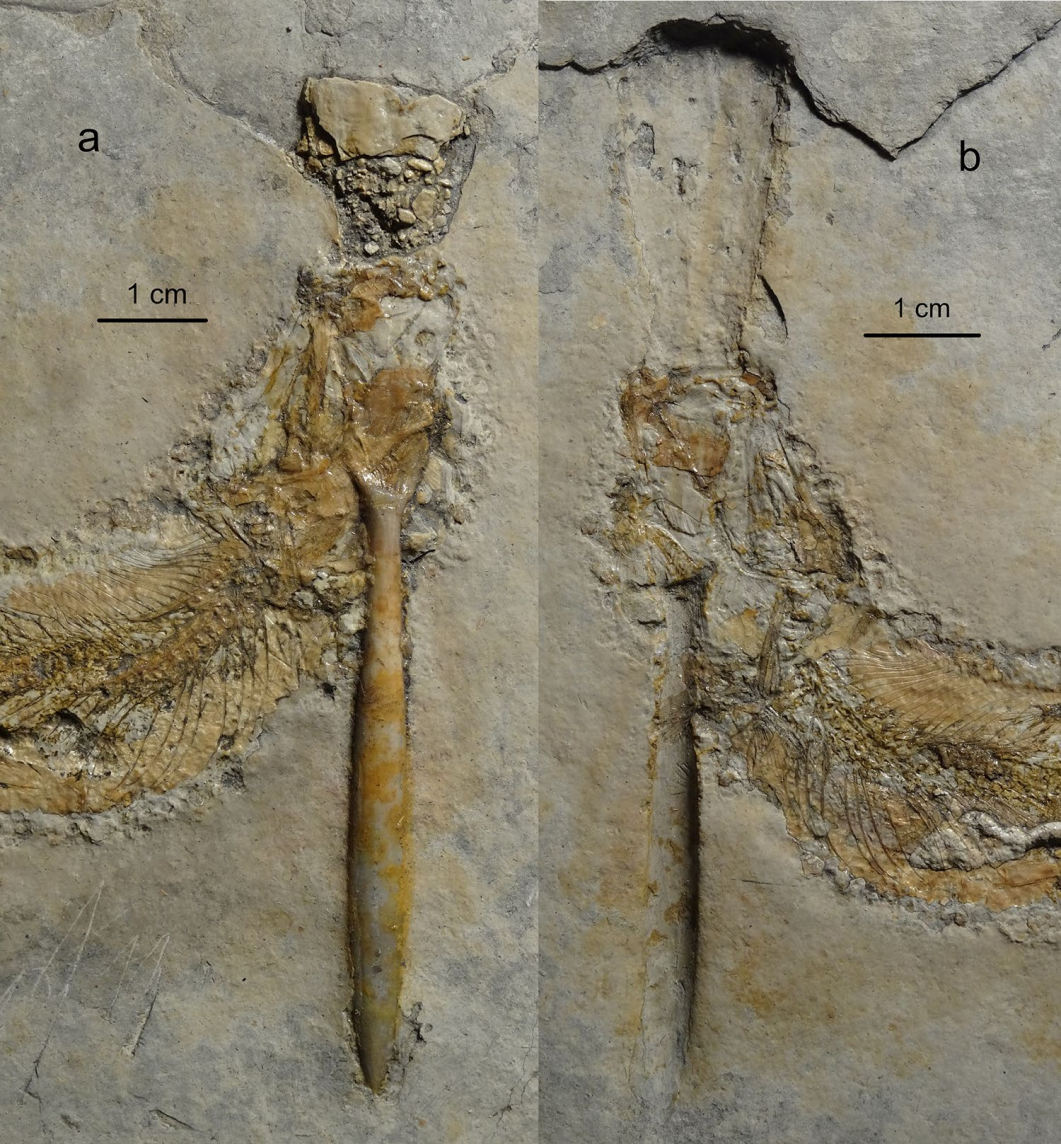
Close-up of the anterior body part of *Tharsis* with belemnite lodged through mouth and gill apparatus (CM4876) from Solnhofen, Bavaria, Germany. (**a**) main slab (**b**) counterpart. (Photo M. Ebert).

The specimen in the collection of the Carnegie Museum in Pittsburgh (USA) is preserved as part and counterpart (Figs. 1 and 2). Specimens of the Carnegie Museum are all labelled “Solnhofen” as locality without distinguishing between the Eichstätt and Solnhofen Basins. In the counterpart it is clearly visible that the fish’s left maxilla is positioned directly over the rostrum of the belemnite (Fig. 2b).

**Fig. 3:**
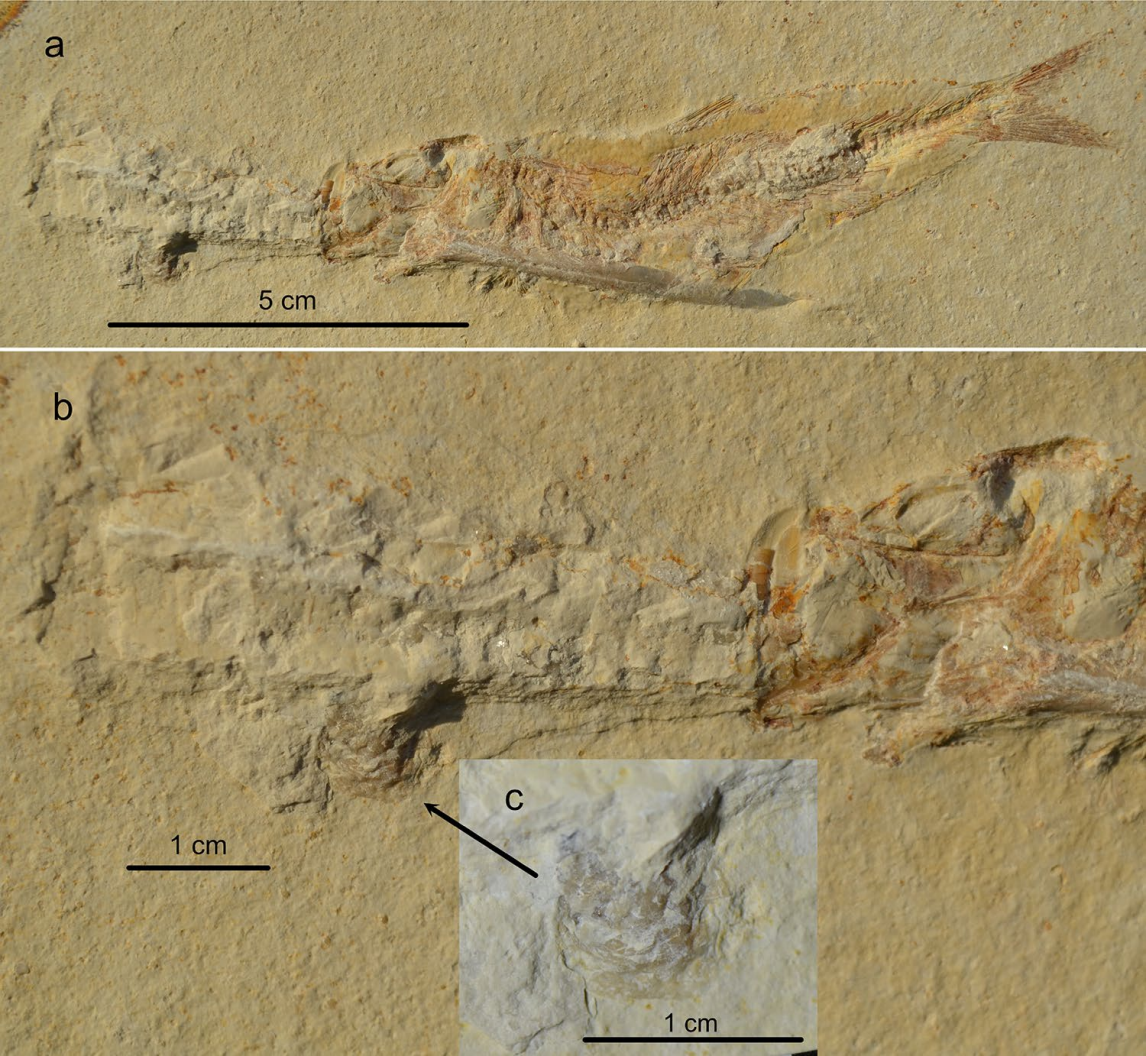
*Tharsis* with belemnite lodged through mouth and gill apparatus (MBH20252251) from Blumenberg, Eichstätt Basin, Bavaria, Germany. (**a**) complete specimen (**b**) close-up of the belemnite phragmocone with an attached oyster (see arrow). (**c**) close-up of the oyster. (Photos M. Ebert).

The specimen in the Museum Bergér (near Eichstätt, Germany; MBH20252251) is remarkable because of the oyster growing on the phragmocone of the belemnite, clearly indicating that the dead belemnite was floating for some time in the water column and that at least the phragmocone was no longer covered by soft tissue at the time of the oyster larvae settling on the carcass (Fig. 3).

**Fig. 4:**
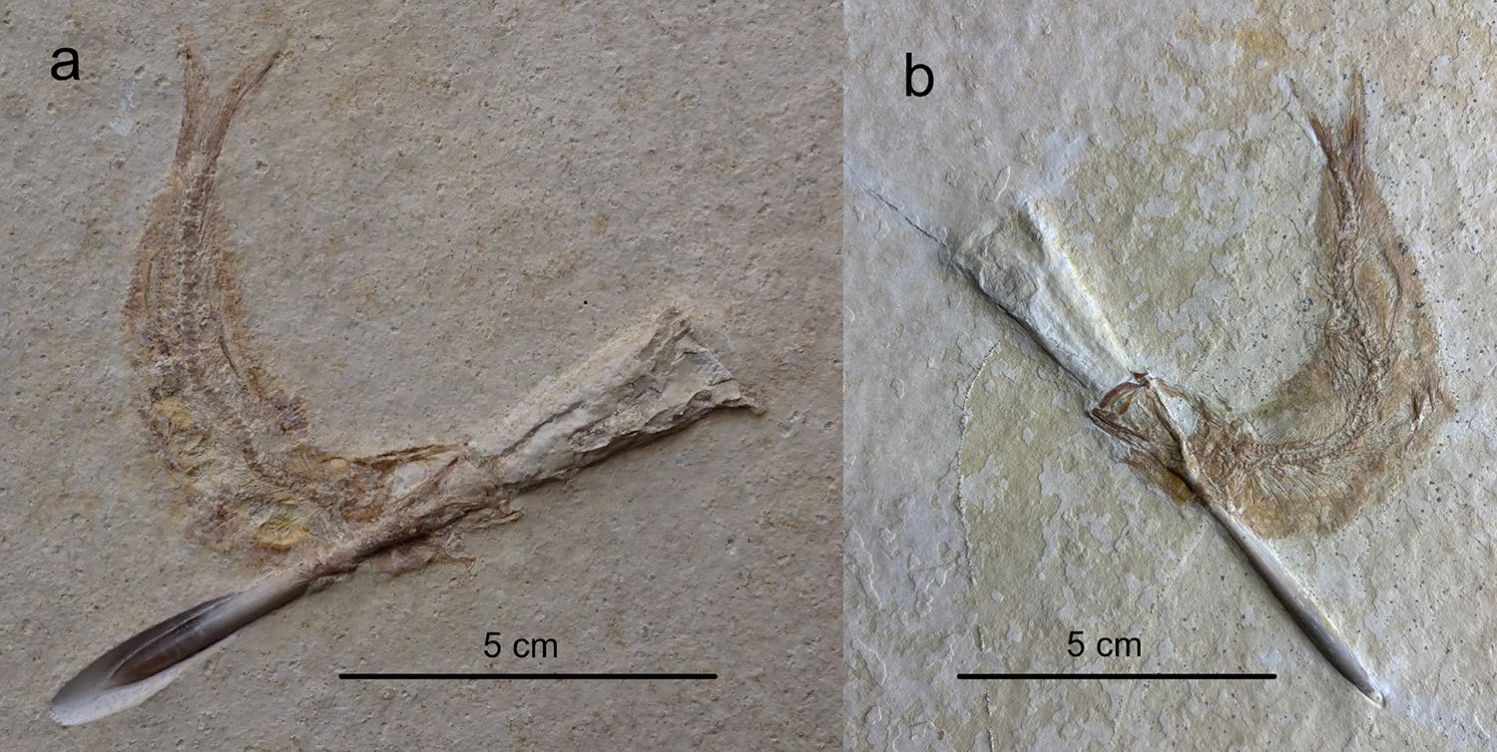
*Tharsis* specimens with belemnite lodged through mouth and gill apparatus from the Solnhofen Archipelago Bavaria, Germany. (**a**) J. Geppert specimen from the Eichstätt or Solnhofen Basin (Photo by J. Geppert and procured with permission by J. Geppert). (**b**) S. Schäfer specimen from the Eichstätt Basin. (Photo by S. Schäfer and procured with permission by S. Schäfer).

We found a third specimen of a *Tharsis* with a belemnite lodged in mouth and gill apparatus from an unknown locality within the Solnhofen Archipelago in the private collection J. Geppert (Fig. 4a).

A fourth *Tharsis* specimen of some 10 cm in length with belemnite in the mouth from the Eichstätt Basin in the private collection S. Schäfer is documented on the fossil collectors’ website *Solnhofen Fossilienatlas* https://www.solnhofen-fossilienatlas.de/fossil.php?fossilid=1713 (Fig. 4b).

## Discussion

For several reasons, it seems highly unlikely that these documented deadly encounters between the fish *Tharsis* and the belemnite *Hibolithes* have been due to “normal” predation of the fish on the belemnite. As mentioned before, the common gut content of *Tharsis* as well as the analysis of its functional morphology points to a micro-carnivore/visual zooplanktivore rather than a habitual predator on belemnites, which compared to the documented subadult *Tharsis* specimens of approximately 10 cm in total length seem far too big anyway. To the contrary, from the Early Jurassic there are several cases reported, where belemnites of similar size ate teleostei of about 10 cm in length^34,35^.

Additionally, the specimen with the attached oyster (Fig. 3) clearly indicates that the belemnite in question was already long dead and devoid of most if not all soft tissue, when the encounter between fish and belemnite took place.

In all specimens, the belemnite entered the *Tharsis*’s mouth with the posterior tip of the rostrum first. The rostrum of a belemnite is smooth, and floating objects in the sea very quickly become overgrown by algae or bacteria^36^. On the fish side of the encounter, a slippery layer of mucus in the throat makes it easy for fish to swallow larger pieces of food^37^.

*Hibolithes*, like many belemnites has a specific shape that may have contributed to the death of the *Tharsis* specimens. The rostrum has a small tip at its posterior end, after which the massive rostrum widens relatively quickly to its maximum thickness (in all studied cases of little less than a centimetre). Then, the rostrum reduces again to about 0.5 cm in diameter at the point where the phragmocone begins. The phragmocone with its gas chambers widens again to more than 2 cm. This shape of the belemnite rostrum with a bulb-shaped end is called *hastate*.

The fully open mouth of the subadult *Tharsis* specimens described here is roughly capable of swallowing prey about 1 cm in diameter. So, if the *Tharsis* swallows the “bulb” of the belemnite rostrum, it can continue sucking it in until the phragmocone widens and is about 1 cm in diameter, but no further, because then it will become too wide for its mouth. Once the first maximum of the rostrum’s “bulb” was overcome, it was probably easier to go further in than out. In addition, most fishes with small teeth capable of suction feeding like *Tharsis* cannot bite off prey or spit out what is far inside^37^.

We do not know, why the belemnites in the available specimens always emerged at the gills and not at least partially migrated into the stomach until it got stuck. However, since entry of the belemnite’s “bulb” into the mouth cavity was an accident rather than intentional and due to the “bulb” was impossible to get rid of through the mouth by reversing the process, the fish may have made the futile attempt to get rid of this strange object through the gills.

Partly swallowed prey is known from other Actinopterygii of the Solnhofen Archipelago as well (*Caturus*^13,15^, *Aspidorhynchus*^17^, *Belonostomus*^11,22,38^ and the Teleost genera *Ebertichthys*, *Orthogonikleithrus* and *Siemensichthys*^11,22^), but in all these cases the prey items are half-swallowed fish, i.e. intended prey albeit too large for swallowing completely. Consequently, parts of the prey fish (in most cases the head) entered the stomach, whereas the posterior body part with the caudal fin still hangs out of the mouth.

Death of the predator probably followed rather quickly. It is known from recent observations that fish suffocate due to large prey stuck in their throats within a few hours at most as the water flow along the gills is disrupted, thus no longer providing sufficient oxygen^13^.

## Conclusions

The evidence suggests that the subadult *Tharsis* specimens have been nibbling and sucking microbial mats or soft tissue remnants off a floating, dead belemnite and accidentally sucked in the “bulb” at the end of the hastate rostrum. Once this happened, the belemnite proved to be a deadly trap due to its peculiar shape and sheer size. Even though the fish tried to pass the obstructive item through its gills, there was no way of getting rid of it, leading to death by suffocation.

## Data Availability

All data generated or analysed during this study are included in this published article.
